# Investigating risk factors that predict a dog’s fear during veterinary consultations

**DOI:** 10.1371/journal.pone.0215416

**Published:** 2019-07-22

**Authors:** Petra T. Edwards, Susan J. Hazel, Matthew Browne, James A. Serpell, Michelle L. McArthur, Bradley P. Smith

**Affiliations:** 1 School of Animal and Veterinary Sciences, University of Adelaide, Roseworthy Campus, Roseworthy, South Australia, Australia; 2 School of Health, Medical and Applied Sciences, CQUniversity Australia, Bundaberg, Queensland, Australia; 3 Department of Clinical Sciences & Advanced Medicine, School of Veterinary Medicine, University of Pennsylvania, Philadelphia, Pennsylvania, United States of America; 4 School of Health, Medical and Applied Sciences, CQUniversity Australia, Adelaide, South Australia, Australia; Memorial University of Newfoundland, CANADA

## Abstract

Attending the veterinary clinic is an integral part of the physical welfare of every companion dog. However, some dogs experience their veterinary visits negatively, which poses a risk of injury to the veterinary staff, their guardian (owner) and themselves. It may also influence the regularity of non-urgent veterinary appointments. To date there have been conflicting reports relating to the proportion of dogs that show fear during their veterinary visits. In this study, we explored the risk factors associated with fear during veterinary examination and in novel situations (including first time at the veterinary clinic) from 26,555 responses in the Canine Behavioral Assessment and Research Questionnaire database. According to their guardians, 41% of companion dogs displayed mild to moderate fearful behaviour when examined by a veterinarian, and 14% exhibited severe or extreme fear. A similar trend was observed with dogs responding fearfully when in unfamiliar situations, including the dog’s first time at the veterinary clinic. Chi-squared tests showed every bivariate relationship between fear and the environmental and demographic factors measured was significant (p < 0.05). The most important predictors of fear in a veterinary examination were, in order: the dog’s breed group (27.1%), their history of roles or activities (16.7%), where they were sourced (15.2%), their weight (12%), the age of other dogs in the household (9.5%) and dog owner experience (6.3%). However, combined these risk factors only explain a total of 7% of variance of fear observed during veterinary examination. This suggests that fear exhibited during veterinary visits is common in dogs, but that the environment or human-animal interactions are likely to contribute more to prevalence and severity of this problem than the demographic factors measured here. We conclude by highlighting opportunities for future research aimed at facilitating less stressful veterinary visits for dogs and their guardians.

## Introduction

Visits to veterinary clinics are integral to maintaining and improving the health and welfare of domestic dogs. However, the veterinary experience can also be stressful for them. It is currently estimated that between 10% and 78.5% of dogs become stressed or fearful in the veterinary clinic [1–14]. For example, Doring et al. [4] identified 13% of dogs refused to enter the veterinary clinic, Stanford [1] reported that 70% of dogs were unwilling to enter, and Mariti et al. [9] found 29% of dogs displayed ‘extreme’ stress in the waiting room, according to their guardians (owners) and a behaviourist assessment. Mariti et al. [11] found guardians reported only 36.4% of 906 dogs tested were calm in the waiting room, while the majority displayed signs of fear, excitement (37.6%) and/or aggression (3.4%). Such disparity in prevalence is likely a reflection of the methodology employed. For example: variation in the behavioural and physiological measures used to assess stress or fear; the person taking the measurement (e.g. investigator, guardian, veterinary nurse, veterinarian); the locations within the veterinary clinic where stress is measured (e.g. waiting room, examination room or kennels and cages); and the context (e.g. guardian present/ absent, mock/real examination). This makes an accurate estimate of the prevalence of stress or fear in dogs visiting veterinary clinics difficult to ascertain.

Negative veterinary experiences can have long-term impacts for the dog, guardian and veterinary staff. A North American study found that the very idea of taking a dog to the veterinary clinic can cause guardians to become stressed (26%) [7]. In fact, many guardians (38%) believe that their dog ‘hates’ going to the veterinarian [7]. As such, guardians want to see their veterinarian interact compassionately with their dog [15], especially when certain methods of handling and restraint can be stressful for animals [16–19]. These attitudes and experiences affect guardian decisions about which veterinarian they see and how often they attend [7]. Further, the behavioural or physiological signs of fear and distress can mirror those of pain, illness and some neurological conditions [20], making accurate diagnoses difficult. Not only can stressed or fearful dogs at the veterinary clinic injure themselves, but they also pose a risk of injury to the veterinary staff and their guardians [20]. Addressing fear at the veterinary clinic and promoting pet-friendly practice is integral to the continual improvement of companion animal welfare.

With the exception of the dog’s sex and age [4], estimated adult weight [3], and the benefit of supportive guardian presence [12], our understanding of dog and guardian characteristics that may exacerbate or ameliorate a dog’s fear or stress response at the veterinary clinic is limited. In order to address this shortfall, we explored the proportion of dogs that show fearful behaviours during veterinary visits according to a large sample of guardians. To do this, we analysed two fear-related, veterinary specific questions and their corresponding behavioural subscales from the Canine Behavioral Assessment and Research Questionnaire (C-BARQ). The C-BARQ is a validated research questionnaire available online to dog guardians [21]. It has been used extensively to investigate factors that influence dog behaviour, personality and temperament in general [22–27], as well as to explore how domestication has influenced behaviour more specifically [28]. The C-BARQ has also been used to measure the behavioural effects of neutering in dogs [29,30], and to investigate the factors associated with aggression [31–33], trainability [34], boldness traits [35], how training can impact dog intelligence [36], how dog behaviour or temperament can influence their health and lifespan [37], and the relationship between dogs and their owners [38]. As such, this extensive dataset of dogs provides an opportunity to build on our understanding of how dogs experience their veterinary care.

## Method

### C-BARQ

C-BARQ contains 100 behavioural items (questions) grouped into 14 subscales (factors) extracted by factor analysis [21]. Guardians respond on a 5-point Likert scale for how serious that behaviour is for their dog or how often it is performed, with ‘0’ being ‘none/ never’ and ‘4’ being ‘extreme/ always’. C-BARQ provides guardians with clear examples of what mild to moderate fear may look like in their dog: “avoiding eye contact, avoidance of the feared object; crouching or cringing with tail lowered or tucked between the legs; whimpering or whining, freezing and shaking or trembling”. Similarly, extreme fear is described as: “exaggerated cowering, and/or vigorous attempts to escape, retreat or hide from the feared object, person or situation”. In this study, C-BARQ responses to two items (questions) related to fear in a veterinary context were analysed to explore the relationship between dog experience at the veterinary clinic, and dog and guardian factors (see Tables 1 and 2 for details). The items asked guardians to report on the extent to which their dog exhibits fearful behaviour during a veterinary examination (Q43; ‘fear of veterinary examination’), and in unfamiliar situations, including examples of first car trip, first time in elevator, first visit to veterinarian, etc. (Q47; ‘fear of unfamiliar’). The two items, fear of veterinary examination and fear of unfamiliar, loaded onto two different behavioural subscales, touch sensitivity and non-social fear, respectively. Touch sensitivity consisted of 4 items (Questions 43, 49, 50 and 51) and refers to dogs that show fearful or wary responses to potentially painful or uncomfortable procedures, including bathing, grooming, nail-clipping and veterinary examinations [21]. Non-social fear contained 6 items (Questions 38, 41, 42, 44, 47 and 48), and refers to dogs that are fearful or wary of sudden or loud noises (e.g. thunder), traffic, and unfamiliar objects and situations [21]. In this study, the predictive value of factors on fear responses in the veterinary context is explored via the two veterinary specific items (Q43 and Q47) in conjunction with the two validated behavioural subscales (touch sensitivity and non-social fear).

**Table 1:** Summary of demographic and environmental covariates and cross-tabulation with mean and standard deviations (SD) of C-BARQ fear variables (with ‘0’ being ‘no fear’, and ‘4’ being ‘extreme fear’).

	Label	N	%	Fear of veterinary examination	Fear of unfamiliar	Touch sensitivity	Non-social fear
				Mean (SD)			
**Sex**	Male	13754	51.79	1.04 (0.010)	1.01 (0.010)	0.76 (0.007)	0.83 (0.007)
	Female	12801	48.21	1.09 (0.011)	1.01 (0.010)	0.76 (0.007)	0.86 (0.007)
**Age**	Puppy (<0.5yr)	583	2.20	0.81 (0.048)	1.02 (0.046)	0.67 (0.031)	0.80 (0.031)
	Adolescent (0.5-3yr)	12033	45.31	1.01 (0.011)	1.05 (0.010)	0.73 (0.007)	0.83 (0.007)
	Adult (>3yr)	13939	52.49	1.12 (0.010)	0.97 (0.009)	0.79 (0.007)	0.86 (0.007)
**Breed group**	Gundogs	4188	15.77	0.79 (0.017)	0.80 (0.015)	0.58 (0.011)	0.68 (0.011)
	Hounds	1481	5.58	1.16 (0.032)	1.05 (0.029)	0.82 (0.021)	0.86 (0.021)
	Mixed Breed/Unknown	7370	27.75	1.33 (0.015)	1.27 (0.014)	0.95 (0.010)	1.04 (0.010)
	Non-Sporting	2138	8.05	1.06 (0.026)	0.99 (0.024)	0.74 (0.017)	0.83 (0.016)
	Terrier	1475	5.55	0.97 (0.030)	0.87 (0.028)	0.74 (0.020)	0.88 (0.020)
	Toys	1986	7.48	1.36 (0.029)	1.10 (0.026)	0.97 (0.020)	0.91 (0.018)
	Utility	3560	13.41	0.74 (0.018)	0.79 (0.017)	0.57 (0.012)	0.66 (0.012)
	Working	4357	16.41	1.00 (0.018)	0.95 (0.016)	0.66 (0.011)	0.77 (0.011)
**Weight**	Smaller (<22kg)	13331	50.20	1.21 (0.011)	1.09 (0.010)	0.87 (0.007)	0.93 (0.007)
	Larger (>22kg)	13224	49.80	0.91 (0.010)	0.93 (0.009)	0.65 (0.006)	0.75 (0.006)
**Neuter status**	No	6460	24.33	0.88 (0.014)	0.88 (0.013)	0.61 (0.009)	0.68 (0.009)
	Yes	20095	75.67	1.12 (0.009)	1.05 (0.008)	0.81 (0.006)	0.90 (0.006)
**Neutered age**	<6 months	16655	62.72	1.05 (0.009)	1.00 (0.009)	0.75 (0.006)	0.83 (0.006)
	6–12 months	4849	18.26	1.12 (0.018)	1.05 (0.016)	0.80 (0.011)	0.88 (0.011)
	12–18 months	1374	5.17	1.05 (0.033)	1.01 (0.030)	0.75 (0.021)	0.85 (0.021)
	>18 months	3677	13.85	1.07 (0.020)	0.99 (0.019)	0.75 (0.013)	0.85 (0.014)
**Reason for neutering**	Birth control	6864	25.85	1.10 (0.015)	1.04 (0.014)	0.79 (0.010)	0.89 (0.009)
	Correct behaviour problems	695	2.62	1.25 (0.051)	1.07 (0.045)	0.87 (0.033)	0.87 (0.029)
	Correct health problems	367	1.38	1.02 (0.063)	0.85 (0.058)	0.70 (0.039)	0.72 (0.040)
	NA	6809	25.64	0.89 (0.014)	0.89 (0.013)	0.62 (0.009)	0.69 (0.009)
	Prevent behaviour problems	796	3.00	1.16 (0.044)	1.04 (0.038)	0.86 (0.029)	0.87 (0.026)
	Prevent health problems	1646	6.20	1.06 (0.030)	0.90 (0.026)	0.74 (0.019)	0.79 (0.017)
	Recommended by veterinarian	1320	4.97	1.22 (0.035)	1.13 (0.032)	0.88 (0.023)	0.95 (0.022)
	Required by breeder	6298	23.72	1.16 (0.016)	1.14 (0.015)	0.84 (0.011)	0.96 (0.010)
	Unknown	1439	5.42	0.98 (0.031)	0.88 (0.028)	0.68 (0.020)	0.80 (0.021)
**Source**	Bred by owner	1083	4.08	0.73 (0.032)	0.76 (0.030)	0.47 (0.020)	0.56 (0.020)
	Breeder	10988	41.38	0.88 (0.011)	0.83 (0.010)	0.63 (0.007)	0.70 (0.007)
	Friend or relative	2451	9.23	1.30 (0.026)	1.16 (0.023)	0.91 (0.017)	0.94 (0.016)
	Other	1420	5.35	1.05 (0.032)	1.00 (0.031)	0.76 (0.021)	0.85 (0.021)
	Pet store	922	3.47	1.37 (0.042)	1.17 (0.038)	0.93 (0.028)	1.05 (0.026)
	Shelter or rescue	8241	31.03	1.22 (0.014)	1.18 (0.013)	0.89 (0.009)	1.00 (0.009)
	Stray	1450	5.46	1.26 (0.034)	1.23 (0.031)	0.92 (0.023)	1.00 (0.021)
**Age when acquired**	Puppy (<0.5yr)	18842	70.95	1.03 (0.009)	0.97 (0.008)	0.74 (0.006)	0.81 (0.006)
	Adolescent (0.5-3yr)	6320	23.80	1.15 (0.016)	1.12 (0.015)	0.82 (0.010)	0.93 (0.010)
	Adult (>3yr)	1393	5.25	1.12 (0.033)	1.06 (0.032)	0.80 (0.023)	0.91 (0.023)
**Health problems**	No	22474	84.63	1.05 (0.008)	1.01 (0.007)	0.75 (0.005)	0.84 (0.005)
	Yes	4081	15.37	1.14 (0.020)	1.01 (0.018)	0.80 (0.013)	0.87 (0.012)
**Role**	Breeding & showing	2247	8.46	0.65 (0.021)	0.74 (0.021)	0.49 (0.014)	0.59 (0.014)
	Field trials / hunting	454	1.71	0.78 (0.050)	0.72 (0.043)	0.55 (0.031)	0.58 (0.030)
	None	18845	70.97	1.18 (0.009)	1.12 (0.008)	0.85 (0.006)	0.94 (0.006)
	Other sports	3545	13.35	0.92 (0.019)	0.76 (0.016)	0.61 (0.011)	0.65 (0.011)
	Working	1463	5.51	0.64 (0.026)	0.65 (0.024)	0.49 (0.016)	0.55 (0.017)
**First dog owned**	No	21728	81.82	1.01 (0.008)	0.99 (0.008)	0.72 (0.005)	0.82 (0.005)
	Yes	4827	18.18	1.30 (0.019)	1.09 (0.016)	0.94 (0.012)	0.95 (0.011)
**Owned dog as child**	No	5281	19.89	1.12 (0.017)	1.02 (0.015)	0.79 (0.011)	0.87 (0.011)
	Yes	21274	80.11	1.05 (0.008)	1.01 (0.008)	0.75 (0.005)	0.84 (0.005)
**Age of other household dogs**	NA	9932	37.40	1.22 (0.013)	1.10 (0.011)	0.87 (0.008)	0.95 (0.008)
	Older	5929	22.33	0.98 (0.015)	1.05 (0.015)	0.69 (0.010)	0.82 (0.010)
	Older and same	479	1.80	1.03 (0.056)	1.07 (0.053)	0.67 (0.037)	0.80 (0.040)
	Older and younger	3001	11.30	0.86 (0.021)	0.86 (0.020)	0.61 (0.014)	0.68 (0.014)
	Older, younger and same	748	2.82	0.83 (0.041)	0.85 (0.040)	0.57 (0.028)	0.63 (0.028)
	Same	1216	4.58	1.11 (0.036)	1.05 (0.033)	0.78 (0.023)	0.89 (0.023)
	Younger	4887	18.40	1.00 (0.017)	0.90 (0.015)	0.74 (0.011)	0.78 (0.011)
	Younger and same	363	1.37	0.99 (0.060)	0.93 (0.057)	0.73 (0.043)	0.76 (0.040)

**Table 2:** Summary of multivariate regression models indicating the predicted severity of fear response (unstandardised Beta coefficients (*B*) and standard error (SE)) by factor during veterinary examination, unfamiliar situations, touch sensitivity and non-social fear.

Factors	Fear of veterinary examination	Fear of unfamiliar	Touch sensitivity	Non-social fear
**Breed group**				
Gundogs	-	-	-	-
Hounds	0.2980* (0.0372)	0.2002* (0.0340)	0.1839* (0.0237)	0.1220* (0.0233)
Mixed Breed/Unknown	0.3217* (0.0261)	0.2747* (0.0238)	0.1927* (0.0166)	0.1697* (0.0164)
Non-Sporting	0.1479* (0.0331)	0.1095* (0.0302)	0.0726* (0.0211)	0.0557* (0.0207)
Terrier	0.0702 (0.0376)	-0.0177 (0.0343)	0.0591 (0.0240)	0.0991* (0.0236)
Toys	0.3823* (0.0355)	0.1508* (0.0325)	0.2309* (0.0226)	0.0871* (0.0223)
Utility	-0.0061 (0.0278)	0.0005 (0.0254)	0.0147 (0.0177)	-0.0036 (0.0174)
Working	0.2244* (0.0268)	0.1747* (0.0245)	0.0901* (0.0171)	0.1026* (0.0168)
**Weight**				
Smaller (<22kg)	-	-	-	-
Larger (> 22kg)	-0.1712* (0.0166)	-0.0927* (0.0151)	-0.1245* (0.0104)	-0.1443* (0.0106)
**Reason for Neutering**				
Birth Control	-	-	-	-
Correct behaviour problems	0.2174* (0.0477)	0.1231* (0.0436)	0.1202* (0.0304)	0.0449 (0.0300)
Correct health problems	0.0647 (0.0642)	-0.0315 (0.0587)	0.0053 (0.0409)	-0.0327 (0.0403)
NA	-0.0593* (0.0219)	-0.0309 (0.0200)	-0.0644* (0.0139)	-0.0854* (0.0137)
Prevent behaviour problems	0.0544 (0.0448)	0.0380 (0.0409)	0.0681 (0.0285)	-0.0135 (0.0281)
Prevent health problems	0.0089 (0.0328)	-0.0765 (0.0300)	-0.0220 (0.0209)	-0.0544* (0.0206)
Recommended by veterinarian	0.0663 (0.0363)	0.0829 (0.0332)	0.0565 (0.0231)	0.0336 (0.0228)
Required by breeder	-0.0405 (0.0236)	0.0067 (0.0215)	-0.0239 (0.0150)	-0.0152 (0.0148)
Unknown	-0.0949* (0.0350)	-0.1256* (0.0319)	-0.0894* (0.0223)	-0.0778* (0.0219)
**Source**				
Bred by owner	-	-	-	-
Breeder	-0.0170 (0.0399)	-0.0043 (0.0365)	0.0361 (0.0254)	0.0274 (0.0250)
Friend or relative	0.2377* (0.0465)	0.1802* (0.0425)	0.2127* (0.0297)	0.1634* (0.0292)
Other	0.0725 (0.0508)	0.0858 (0.0464)	0.1150* (0.0324)	0.1262* (0.0319)
Pet store	0.2146* (0.0567)	0.1687* (0.0517)	0.1598* (0.0361)	0.2220* (0.0355)
Shelter or rescue	0.1633* (0.0443)	0.1635* (0.0405)	0.1780* (0.0282)	0.1891* (0.0278)
Stray	0.1710* (0.0519)	0.1719* (0.0474)	0.1955* (0.0331)	0.1762* (0.0326)
**Role**				
Breeding & showing	-	-	-	-
Field trials /hunting	0.1254 (0.0623)	-0.0151 (0.0569)	0.0503 (0.0397)	-0.0347 (0.0391)
None	0.2551* (0.0300)	0.1867* (0.0274)	0.1489* (0.0191)	0.1370* (0.0188)
Other Sports	0.1324* (0.0337)	-0.0693 (0.0308)	0.0131 (0.0215)	-0.0588* (0.0211)
Working	-0.0844 (0.0415)	-0.1612* (0.0379)	-0.0563 (0.0265)	-0.1265* (0.0261)
**First dog owned**				
No	-		-	
Yes	0.1622* (0.0203)		0.1166* (0.0130)	
**Age of other household dogs**				
Single dog	-	-	-	-
Older	-0.1328* (0.0209)	0.0190 (0.0185)	-0.0925* (0.0133)	-0.0660* (0.0127)
Older and same	-0.0813 (0.0571)	0.0266 (0.0519)	-0.1157* (0.0364)	-0.0855 (0.0356)
Older and younger	-0.1858* (0.0265)	-0.0971* (0.0238)	-0.1172* (0.0169)	-0.1462* (0.0163)
Older, younger and same	-0.2221* (0.0476)	-0.1202* (0.0432)	-0.1456* (0.0303)	-0.1858* (0.0297)
Same	-0.1273* (0.0369)	-0.0589 (0.0336)	-0.0919* (0.0236)	-0.0747* (0.0231)
Younger	-0.1425* (0.0214)	-0.1220* (0.0194)	-0.0706* (0.0136)	-0.1137* (0.0134)
Younger and same	-0.1945* (0.0641)	-0.1537* (0.0585)	-0.1028 (0.0409)	-0.1593* (0.0401)
**Constant (Intercept)**	0.7507* (0.0531)	0.7526* (0.0483)	0.5643* (0.0338)	0.7213* (0.0331)
N	25,093	25,093	25,093	25,093
*R^2^* (%)	7.06	5.45	8.58	8.24
Adjusted *R^2^*	6.94	5.32	8.46	8.12
Residual Std. Error	1.1717 (df = 25058)	1.0707 (df = 25059)	0.7470 (df = 25058)	0.7354 (df = 25059)
F Statistic	56.0120* (df = 34, 25058)	43.7294* (df = 33, 25059)	69.1927* (df = 34, 25058)	68.2073* (df = 33, 25059)

*p<0.01; standard errors are given in brackets after unstandardised Beta coefficients.

All factors dummy coded (0,1); unstandardised beta coefficients (*B)* reflect mean differences from the reference category. All dependent variables scaled from 0 (no fear) to 4 (extreme fear)

### Dog sample

Retrospective data was collected from guardians completing C-BARQ online (https://vetapps.vet.upenn.edu/cbarq/) between 2005 and 2016. Responses that did not include answers to the vital items–Question 43 (fear of veterinary examination) and Question 47 (fear of unfamiliar situations)–were excluded. Breeds were then categorised into breed groups via the Australian National Kennel Council (ANKC) breed list. In the event that a breed was unrecognised by the ANKC, it was categorised in accordance with the American Kennel Club (AKC) (e.g. American Eskimo dog, rat terrier, great Pyrenees, chinook, Spanish water dog). Some breeds were identified in ANKC by a different name (e.g. Belgian sheepdog as groenendael; English bulldog as British bulldog). Breeds not recognised by the ANKC, AKC or the Fédération Cynologique Internationale (FCI) breed lists (English shepherd and American pit bull terrier), were removed. Dogs listed as crossbreeds or of unknown breed were included as ‘mixed breed’. Only dog breeds with a minimum of 50 responses were included. Dog age, neuter age, and age of acquisition were converted into years, and dog weight converted to metric (lbs to kg). C-BARQ is available for guardians with dogs six months and over, and as such, ‘puppies (<6 months)’ in this study refer only to dogs that were six months of age at the time of evaluation (N = 583). This research was considered ‘negligible risk’ as it involved the use of an existing collection of non-identifiable data and there was no foreseeable risk of harm or discomfort. As such, it was granted a formal waiver of ethics approval from The University of Adelaide Human Research Ethics Committee review.

### Statistical analysis

Cross-tabulations were performed using the R statistical programming environment [39] between all independent variables and the outcome measures, fear of veterinary examination and fear of unfamiliar. Chi-square tests revealed that every bivariate relationship between fear response and environmental and demographic variables were significant, due to the large sample size. Accordingly, our analyses focused on determining the magnitude of effect sizes, and assessing the relative importance of each variable in predicting fear of veterinary examination and fear of unfamiliar, in both a univariate and multivariate context. In the first step, we assessed relative importance using two metrics within a framework called *dominance analysis* [40, 41]. The first metric is calculated by entering each variable in isolation (e.g. simple regression), and expressing each model *R*^2^ relatively: e.g. as a percentage of the sum of *R*^2^ explained by all models. The second metric, *lmg* [42], considers the *R*^2^ over all possible combinations of predictors: e.g. given predictors X1, X2, X3, models y ~ X1, y ~ X2, y ~ X3, y ~ X1 + X2, y ~ X2 + X3, y ~ X1 + X3, y ~ X1 + X2+ X3 are considered. Thus, a single predictor’s relative contribution is assessed in terms of the drop in *R*^2^ when it is removed, in all possible multivariate contexts. In the second step, two multiple regression models were fitted for fear of veterinary examination, fear of unfamiliar, touch sensitivity and non-social fear. The first model included all predictors (‘all-in’ regression model). The second model included only those predictors that contributed more than 5% of the explained variance according to the *lmg* importance metric (‘parsimonious’ regression model). Though 5% is an arbitrary threshold for inclusion, in contrast to stepwise methods the *lmg* criteria is a robust variable selection method, because the entire space of possible regression models is evaluated [43]. A Spearman rank-order correlation between fear of veterinary examination and fear of unfamiliar, and the two subscales touch sensitivity and non-social fear was conducted to ascertain the overlap between individual dogs.

## Results

The sample of 26,555 valid responses was evenly distributed by dog sex (51.79% male; Table 1), and the mean dog weight was 22.78kg (±14.57kg; median 21.60kg). The mean dog age was 4.52 years (±3.49 years; median 4.00 years), mean neuter age was 0.84 years (±1.55 years; median 0.48 years) and mean age acquired was 0.77 years (±1.49 years; median 0.21 years). The majority of dogs were healthy (84.63%), neutered (75.67%), and purchased as companions (70.97%), with no specific sporting or working role. The most common reasons for neutering were birth control (25.85%), and required by breeder (23.72%). Dogs were most commonly acquired from a breeder (41.38%) and shelter or rescue (31.03%), while the least common source for dogs was a pet store (3.47%), followed by those bred by their guardians (4.08%). The majority of guardians were experienced dog owners, having had dogs previously as adults (81.82%) and/or as children (80.11%). Just over half of dogs (62%) were from multi-dog households. Mixed breeds or dogs of unknown breed were the most commonly reported (27.75%), followed by working breeds (16.41%) and gundogs (15.77%).

Of the 26,555 dogs, 41.02% exhibited mild to moderate fearful behaviour when examined by a veterinarian, and a total of 14.23% of all guardians reported that their dog showed severe or extreme fear during veterinary examination. That is, over half (55.25%) of all dogs showed fear in some capacity in a veterinary context. Similarly, 46.68% of dogs showed mild-moderate signs of fear in new situations, including potentially the first visit to the veterinary clinic, while 11.02% of all dogs displayed severe or extreme fear in unfamiliar situations. In contrast, just over a third of dogs displayed at least some form of fear (mild-extreme) for the corresponding subscales of touch sensitivity and non-social fear (35.01% and 37.00% respectively). The correlation between fear of veterinary examination and fear of unfamiliar was 0.45 (*p* < 0.001), and between the two subscales touch sensitivity and non-social fear was 0.44 (*p* <0.001). The mean score of fear for both items (fear of veterinary examination and fear of unfamiliar) and both subscales (touch sensitivity and non-social fear) for each of the independent variables are displayed in Table 1.

### Multivariate regression of fear response

Linear regression models were used to explore the predictive importance of dog and guardian factors in determining fear of veterinary examination and fear of unfamiliar. Table 2 highlights the relationships between the different environmental and demographic factors predicting fearful behaviour, analysed through parsimonious regression models for fear of veterinary examination, fear of new situations, touch sensitivity and non-social fear. Considering ‘all in’ analyses yielded only a slight increase in explained variance of fear responses in comparison to parsimonious models, we focus on the latter. The parsimonious models were significant for all dependent variables, explaining 7.06% of variance in fear of veterinary examination (F = 56.01, df = 34, 25058, p< 0.01), 8.58% of variance in touch sensitivity subscale (F = 69.19, df = 34, 25058 p < 0.01), 5.45% of variance of fear of unfamiliar (F = 43.73, df = 33, 25059, p < 0.01), and 8.24% of variance of non-social fear subscale (F = 68.21, df = 33, 25059, p < 0.01). This effect size refers to the proportion of the variation in fearful behaviour that can be attributed to the factors discussed in the following section. For example, approximately 7.06% of the variation of fear observed during veterinary examinations can be attributed to these factors. Likewise, these factors account for 5.45% of the variation in fearful behaviour observed in unfamiliar situations. The constant (intercept) represents the grand mean score of fear response for all dependent variables (fear of veterinary examination, fear of unfamiliar, touch sensitivity and non-social fear) for all referents (e.g. gundogs or hounds within breed group). The referent score (unstandardised beta coefficient; *B*) follows the same scale used when guardians reported on each item in C-BARQ, where a score of ‘0’ equates to ‘no fear’, and ‘4’ represents ‘extreme fear’. A dog’s predicted fear response score is calculated with the equation: B*1 + Constant. As such, coefficients reflect adjustments to the conditional mean, given each of the predictors.

### Relative importance of factors in explaining variation of fear

The relative importance of each of the factors in predicting fear of veterinary examination, fear of unfamiliar, touch sensitivity and non-social fear are shown in Table 3. While both bivariate (‘first’) and multivariate (‘*lmg*’) analyses are displayed, only the multivariate results are discussed here as both models demonstrate a similar pattern of effects. Fourteen variables explained more than 5% of the variation in fearful behaviour observed, and are listed in descending order of importance. Only those factors that can be assigned to over 5% of the effect size observed are discussed. A dog’s breed group was the strongest predictor of fear of veterinary examination (27.14%), fear of unfamiliar (26.98%) and touch sensitivity (23.15%). Non-social fear was the only scale in which both role of the dog (24.35%) and dog source (20.02%) explained more of the variance of fear than breed group (18.70%). Role of the dog, dog source, weight and age of other dogs in the household were important factors across all scales. The reason for neutering contributed to the variance of fear observed in all scales, except fear of veterinary examination, while whether the guardian had owned dogs before was only important in fear of veterinary examination and touch sensitivity. Overall, these factors were significant in predicting fear responses in a veterinary context and are important in identifying how dogs experience their veterinary care.

**Table 3:** Relative variable importance (%) in predicting fearful behaviour from C-BARQ items fear of veterinary examination and fear of unfamiliar and subscales touch sensitivity and non-social fear in a bivariate (‘first’) and multivariate (‘*lmg*’) context. *lmg* scores that indicate the variable captures more than 5% of explained variance in fearfulness are discussed in text.

	Fear of veterinary examination		Fear of unfamiliar		Touch sensitivity		Non-social fear	
Predictor %	first	*lmg*	first	*lmg*	first	*lmg*	first	*lmg*
Breed group	24.26	27.14**	24.55	26.98**	21.77	23.15**	18.59	18.70*
Role	17.36	16.68*	23.34	26.81*	17.40	16.79*	21.16	24.35**
Source	16.93	15.21*	21.64	19.70*	17.99	17.40*	20.80	20.02*
Weight	10.35	11.97*	4.64	5.20*	10.81	14.38*	7.24	10.14*
Age of other household dogs	8.93	9.51*	6.81	6.99*	8.66	8.62*	8.69	10.31*
Neuter reason	6.10	4.47	8.34	6.68*	7.80	5.89*	9.56	6.59*
Neuter status	5.18	2.28	4.60	2.25	6.58	3.53	7.85	4.56
First dog owned	6.32	6.33*	1.43	1.00	6.33	6.84*	2.15	1.65
Age when acquired	1.25	0.81	3.16	2.05	1.08	0.89	2.87	1.80
Age	1.96	3.94	1.13	1.83	0.82	1.50	0.24	0.59
Neutered age	0.34	0.31	0.31	0.39	0.31	0.48	0.36	0.43
Health problems	0.36	0.50	0.01	0.05	0.24	0.32	0.08	0.15
Sex	0.21	0.53	0.00	0.03	0.00	0.04	0.26	0.59
Owned dog as a child	0.46	0.32	0.05	0.03	0.22	0.16	0.15	0.12

** The largest predicting factor

* Factors that contribute over 5% to the variance observed in fear responses to veterinary examination, new situations, non-social fear and touch sensitivity, and are discussed in text

#### Breed group

Breed group was the largest predictor of fearful behaviour at the veterinary clinic (Table 3). Relative to the other breed groups, Table 2 shows toy breeds (*B* = +0.38), mixed breeds (*B* = +0.32) and hounds (*B* = +0.30) predicted the highest scores of fear when examined by a veterinarian. The utility (*B* = -0.01) and gundog (*B* = 0) groups exhibited the least fear during veterinary examination. The same breed group patterns are observed in the corresponding touch sensitivity subscale. However, when assessing fear of unfamiliar situations, mixed/unknown breeds (*B* = +0.28), hounds (*B* = +0.20) and working dogs (*B* = +0.18) displayed the highest scores of fear, while terriers (*B* = -0.02) and gundogs (*B* = 0) exhibited the least fear in new situations. The highest levels of non-social fear were observed in mixed breeds (*B* = +0.17), and hounds (*B* = +0.12), while the lowest non-social fear scores were displayed by utility (*B* = -0.004) and gundogs (*B* = 0).

#### A dog’s employment or activity history

The activities or roles a dog has been involved in are the second largest predictor of fear of veterinary examination (16.68%) and fear of new situations (26.81%), and the most important predictor of non-social fear (24.35%; Table 3). Relative to all roles or activities (Table 2), dogs used for breeding and showing (*B* = 0) and dogs with a working background (*B* = -0.08) predicted the lowest scores of fear when examined by the veterinarian. Conversely, companion dogs (with no history of formal roles or activities) predicted the highest scores of fear when examined by a veterinarian (*B* = +0.26). Dogs involved in other sports (*B* = +0.13), and field trials or hunting (*B* = +0.13) also tended to exhibit more fear during veterinary examination than working dogs. The same trend was observed in the corresponding touch sensitivity subscale, with companion dogs displaying the highest scores of fear (*B* = +0.15), and those in working roles the least fear (*B* = -0.06). Similarly companion dogs were likely to exhibit the highest fear responses in new situations (*B* = +0.19), and non-social fear (*B* = +0.14), while again, dogs in working roles showed the least fear (*B* = -0.16; *B* = -0.13 respectively).

#### Source of the dog

The source of the dog was also a large predictor of fear response across all dependent variables (Table 3). Dogs acquired from a breeder (*B* = -0.02) or bred by their guardians (*B* = 0) predicted the lowest fear scores when examined by a veterinarian (displayed in Table 2). Whereas, dogs acquired from a friend or relative or purchased from a pet store predicted the highest fear scores (*B* = +0.24; *B* = +0.22 respectively). Dogs acquired from a friend or relative were also likely to have higher scores in the touch sensitivity scale (*B* = +0.21), followed by those acquired as a stray (*B* = +0.20), those from a shelter or rescue (*B* = +0.18) and those from a pet store (*B* = +0.16). A slightly different trend is observed in fear of new situations and non-social fear. Dogs acquired from a friend or relative (*B* = +0.18), or as a stray (*B* = +0.17) displayed the highest scores of fear in new situations. In contrast, the highest non-social fear was exhibited by dogs purchased from a pet store (*B* = +0.22), followed by those from a shelter or rescue (*B* = +0.19). Dogs purchased from a breeder were the least fearful of unfamiliar situations (*B* = -0.004), while dogs bred by their guardian had the lowest touch sensitivity and non-social fear scores (*B* = 0).

#### Weight

A dog’s size also contributed over 5% of the variation observed in fear response (Table 3). Larger dogs (>22kg) exhibited lower fear scores in comparison to smaller dogs (<22kg) when examined by a veterinarian (*B* = -0.17) and in new situations (*B* = -0.09), and had lower touch sensitivity (*B* = -0.13) and non-social fear (*B* = -0.14; Table 2).

#### Age of other household dogs

The ages of other dogs in the home also influenced a dog’s fear response (Table 3). Dogs living without conspecifics displayed the most fear across all dependent variables (*B* = 0; Table 2), except fear of unfamiliar where dogs living with older dogs (*B* = 0.01), or dogs older and the same age (*B* = 0.03) predicted slightly higher fear in that context. Conversely, dogs that lived with other dogs that were older, younger and the same age (e.g. living with at least three other dogs) showed the lowest scores of fear during veterinary examination (*B* = -0.22) and in the touch sensitivity (*B* = -0.15) and non-social fear (*B* = -0.18) subscales. Whereas, dogs that lived with others that were younger and the same age showed the least fear in new situations (*B* = -0.15).

#### Other contributing factors

The reason a dog was neutered also contributed to the variance of fear observed across the majority of variables, but did not contribute over 5% of variance toward fear of veterinary examination (Table 3). Dogs neutered in order to correct behaviour problems exhibited the highest scores of fear in new situations (*B* = +0.12; Table 2), touch sensitivity (*B* = +0.12) and non-social fear (*B* = +0.05). Conversely, dogs neutered for unknown reasons displayed the lowest scores of fear in new situations (*B* = -0.13) and in touch sensitivity (*B* = -0.09). Lastly, the guardian’s experience in owning a dog predicts a small proportion of fear observed during veterinary examinations and in touch sensitivity (Table 3). First time dog owners had dogs that exhibited the highest scores of fear during veterinary examination (*B* = +0.16; Table 2), and in touch sensitivity (*B* = +0.12), in comparison to guardians that had owned dogs previously.

## Discussion

A large sample size of companion dogs was used to explore the proportion and characteristics of dogs that show a fearful response when visiting a veterinary clinic and in unfamiliar situations. According to their guardians, 41% of dogs experienced mild to moderate fear when examined by the veterinarian, while one in seven dogs (14%) exhibited severe or extreme fear in the same context. Likewise, 47% of companion dogs exhibited mild to moderate fear in new situations, including the first time at the veterinary clinic, while 11% exhibited severe-extreme fear. These figures fall within the broad estimates of previous cross-sectional studies [1, 2, 4, 5, 8–12, 14] and arguably provide a more realistic rate of global prevalence of fear of the veterinarian in dogs. In contrast, the touch sensitivity and non-social fear subscales demonstrated a smaller proportion of companion dogs exhibiting fear in some capacity (35% and 37% respectively).

The individual items likely measure a wide range of fearful behaviours in dogs visiting a veterinarian as they correlated with different behavioural subscales (touch sensitivity and non-social fear). This is supported by the positive moderate correlation between the two items (fear of veterinary examination and fear of unfamiliar) and the two subscales (touch sensitivity and non-social fear). It suggests that while there is some overlap across dependent variables, each individual dog’s fear response differs slightly according to context. Fear of veterinary examination likely reflects the association made with handling and potentially painful experience in a clinical setting, while fear of unfamiliar situations (including first time at the veterinary clinic) could reflect a generalised neophobic response. The reduced prevalence of fear in the subscales (in comparison to the two individual items), indicates the more general nature of touch sensitivity and non-social fear. That is, while the subscales include items referring to a dog’s veterinary experience, the scales also contain other items that do not. As such, the higher prevalence of fear observed in the individual items reflects the many factors within the veterinary context that may be the cause or catalyst of that fear.

The prevalence of fear in a veterinary context may be influenced by a dog’s genetic predisposition to fear [44]. For example, Godbout et al. [3] identified a small proportion of puppies (10%) that displayed extreme avoidance behaviours during a mock examination. They suggest this likely reflects a proportion of dogs that exhibit anxious behaviours through to adulthood, as a result of a genetic predisposition to an anxious temperament. This is an important area for future research and our results indicate a similar proportion of dogs with severe fear.

As a group, all predictors explained between 5 and 7% of variance in fear of unfamiliar and fear of veterinary examination. The most important predictors were, in order, the dog’s ANKC breed group, the dog’s employment or activity history, where they were sourced, their weight, the age of other dogs in the household, reason for neutering and guardian’s level of experience of dog ownership. The low effect size (i.e. 7% for fear of veterinary examination) suggests these factors combined set the foundation of a dog’s predisposition to fearful experience in the veterinary clinic, while other influences (e.g. environmental, previous experience or human-animal interactions) pinpoint the severity of the fear response. This mirrors previous studies investigating neuter age and stranger-directed aggression [29] or fear-related behaviours [30], source of acquisition and non-social or stranger-directed fear [45], and litter size and personality [46]. Indeed, Casey et al. [47] suggest the factors associated with human-directed aggression explain a similar amount of variance (<10%), and emphasise that individual experience is likely of much greater importance in determining behaviour. Thus, while these risk factors are invaluable in helping inform opinion on how a dog may respond in a veterinary clinic, we emphasise the importance for veterinary staff to take active steps to prevent negative experience from developing in the first place. Edwards et al. [48] provides a summary of current strategies thought to reduce or prevent distress in the veterinary clinic, while Dawson et al. [49] have developed a canine and feline welfare assessment tool that can assist clinics in determining their overall score for pet-friendly practice.

A dog’s breed is frequently attributed to variance in behaviour, and as such, it may be unsurprising that breed group was the best predictor of fear across all the dependent variables. Blackwell et al. [50] reflect that mixed breeds, according to their guardians, were generally more likely to be fearful of noises in comparison to other breed groups. In contrast, in a cross sectional study, dogs in utility and hound groups were more aggressive to family members than mixed breeds [47]. Although this may simply highlight the difference between aggression and fear, it suggests context is important in determining behaviour–the same dog may react fearfully to an unexpected noise but aggressively toward an unfamiliar person. Additionally, some individual breeds (e.g. dachshunds, Chihuahuas or Jack Russell terriers) have been associated with an increased likelihood of showing aggression toward their guardians and strangers [31]. However, comparison across the literature is difficult due to unstandardised methods (e.g. subjective survey or objective experimental design), breed definitions (e.g. conventional breed grouping or genetic cluster) and behaviour or trait analysed [51]. Breed-specific behaviour then, likely varies from a combination of genetics, early experience and the current environment; nature via nurture [51–53]. Indeed, breed differences may simply reflect features the breed has been specifically selected for (and not all aspects of behaviour), and emphasise the impact of early experience for breeds typically raised in different environments [54]. It is also important to note that while it may provide a predisposition to fear, temperament emerges early in development and remains relatively stable across situations and over time [55]. In contrast, fear of veterinary care likely incorporates a learnt component as dogs can associate adverse veterinary experiences with the veterinary clinic, and are learning to anticipate the negative experience in subsequent visit [4, 56]. Dawson et al. [49] suggests there is considerable variation between veterinary clinics and their practice or approach to animal welfare. Therefore it stands to reason that where the approach differs, so does the dog’s fear response. Considering a dog’s ANKC breed group explained the largest proportion of variance of fear observed, extending the veterinary consult, or providing extra support to guardians of specific breeds may be valuable in reducing fear in the veterinary clinic. However, active steps should be taken to prevent negative experience in the first place regardless of breed.

In the present study, dogs previously employed in working, breeding and showing roles had lower fear of the veterinarian and fear of unfamiliar, while companion animals were most likely to show high levels of fear in the same contexts. This is supported by dogs in these roles also having the lowest touch sensitivity scores. The roles that dogs are employed in can influence aspects of their personality and behaviour, further highlighting the contention between inter- and intra-breed variation in behaviour. For example, Lofgren et al. [26] argues that Labrador retrievers purchased as companions or employed in a gundog role showed higher human and object fear than Labradors that were show dogs, while companion Labradors exhibited greater noise fear than those that were gundogs or show dogs. The reduced risk of fear of veterinary exam and unfamiliar in dogs employed in breeding or working roles may reflect an increased familiarity with procedures associated with veterinary care (i.e. grooming, handling or restraint). As such, we suggest that appropriate handling and grooming practice for companion dogs is equally as important as basic manners training and socialisation in reducing fear in the veterinary context.

The source of acquisition of the dog was also a predictor of fear response in a veterinary context. Dogs acquired from friends or relatives, pet stores shelters and rescues, or as strays were most likely to be fearful during veterinary visits or have high touch sensitivity and non-social fear. In contrast, those bred by their guardian exhibited less fearful behaviour. This reflects a similar finding by Blackwell et al.[50], that dogs bred by their guardians are less likely to show fear responses to noises than dogs from other sources. Further, puppies from pet stores had increased risk of behavioural issues in comparison to puppies purchased from breeders [45, 57], while the quality of maternal care can have long term behavioural fallout and alter the physiological responses to stress [44,58]. That is, dogs bred by their guardians may reflect a higher level of both maternal and guardian care and/or appropriate socialisation and early experience in the first few weeks of life in situations where guardians know they are keeping a puppy from a litter. In addition, it may also reflect a negative influence of transitioning to new homes in general, or more specifically, a negative experience *while* transitioning to a new home (i.e. plane travel, long distance car rides, lack of familiarity, early separation or being unfamiliar human contact). Indeed, guardians reported a significantly higher risk of destructiveness, excessive barking, fearfulness, reactivity to noises, resource guarding and attention-seeking behaviour in dogs that were separated from their litter before six weeks of age in comparison to those separated at eight weeks [59]. Therefore, the puppy’s experience for the first several weeks of life requires careful consideration in future investigation of how dogs experience their veterinary visits. Veterinary staff and guardians alike can capitalise on the impact of this critical early period by limiting early negative experience and maximising early positive experience in the veterinary clinic.

Dog size also influences the overall variation of fear observed during veterinary examinations, with lighter (and therefore generally smaller) dogs (<22kg) predicting higher fear scores than heavier dogs (>22kg). Smaller dogs have also been found to be more vocal during observation on the floor of the veterinary clinic than larger dogs [3], and are associated with aggressive and excitable, and anxious and fearful behaviour in comparison to larger dogs [60]. Guardians of small dogs behave differently when it comes to allowing off lead play or socialisation in comparison to guardians of large dogs [61], and so it is possible that smaller dogs are treated differently when it comes to handling or grooming practice in comparison to their larger counterparts. Alternatively, the majority of dogs exhibit fear-related behaviour when examined on the examination table [4], so it is likely that the greater fear response observed for smaller dogs simply reflects a fear of the examination table. Either way, one way to reduce fear may be to examine dogs (of all sizes) on the floor or where they are most comfortable. It must be noted however, that the statistical models estimated the effect for breed when controlling for size and vice versa. As such, the extent to which each of the factors contribute to fear of veterinary examination and fear of unfamiliar individually is unknown, and likely inseparable, considering artificial selection for breed phenotype includes size.

Dogs living in single dog households were likely to exhibit higher fear in almost every scenario (fear of veterinary exam, touch sensitivity and non-social fear), while dogs living with others older, younger and the same age were the least fearful in those same contexts. The different trend observed in fear of unfamiliar may reflect the range of situations the question proposes, of which fear during the first time at the veterinary clinic is only one. While it may be simpler to compare singleton to multi-dog households, we emphasise that there was a significance effect of ages of other dogs in the household, and not simply living with conspecifics, that predicted lower fear in the veterinary context. This highlights the complexity involved in the social dynamic of living with other dogs *and* the nuance of different ages other dogs have on veterinary experience, relative to single dog homes. Perhaps it’s the ages of other dogs in the home that influences social learning–younger dogs may learn from older dogs via observation, or dogs of the same age by participation. Living with multiple dogs of different ages may provide the social cues for confidence relating to fear during veterinary examination, or alternatively greater resilience generated by unpredictable and frequent social interaction observed within a multi-dog household. While dog age was not a predictor of fear relative to other factors in the present study, Doring et al. [4] identified dogs under two years of age exhibiting less behavioural signs of fear in a veterinary context than middle-aged or older dogs. As with the benefit of a positive guardian presence in reducing fear [12], dogs that attend the veterinarian with another familiar, younger (< 2 years) and confident dog may take their social cues from that dog and be less fearful. It is unclear however, whether all dogs in the home attend the veterinarian together (and whether they show similar fear responses), or whether a reduced risk of fear at the veterinary clinic results from some social interaction that occurs at home. As such, the true impact of this factor as a predictor of fear in the veterinary context is difficult to discern. It is likely though, that guardian or conspecific presence is beneficial, but conditional on the type of attachment [62]. Further investigation into the dynamic nature of such relationships and in which environment (e.g. home or veterinary clinic) they are most effective in is required.

Guardian level of experience was another contributing factor for fear of veterinary examination and touch sensitivity. Guardians that had never owned a dog previously were more likely to have dogs that exhibited higher fear responses. While over 25% of guardians are able to identify obvious signs of stress in dogs [63], Flint et al. [64] suggest a lack of experience in dog behaviour or attendance at dog training classes is associated with guardians being less likely to identify fear correctly. This suggests a potential for fear to be under-reported in dogs with inexperienced guardians. It also constitutes a significant risk to companion animal welfare as accurately recognising fear is essential in reducing fear in the veterinary context [56, 65–68]. Further, while responses from single-dog households may also correlate with first-time dog guardians, the social dynamic involved in dogs living with other dogs of varying ages outweighs guardian experience when predicting fear during veterinary visits. Overall, the very real value of both guardian experience and age of other dogs in the household highlight the importance of guardian education focusing on canine socialisation and body language to increase their ability to accurately identify overt signs of stress as a minimum.

Guardians that neutered their dog in order to correct behaviour problems had dogs that were more fearful in new situations, and had higher touch sensitivity and non-social fear. This is supported by Lind et al. [14] who found that dogs with guardian reported behaviour problems were also rated as more stressed during veterinary visits by the guardian, and the (blinded) researcher. However, the cross-sectional design of the present study, and that of Lind et al. [14] is limited in that it may reflect dogs that already have behavioural problems, and hence are neutered, are then more likely to be touch sensitive and have non-social fear. Further longitudinal studies are necessary to investigate the influence of neutering on dog behaviour.

While the C-BARQ is a validated questionnaire that clearly describes the behaviour of interest, it is vulnerable to measurement errors, including: conservative reporting (if guardians are predisposed to report on items like problem behaviours optimistically); guardian interpretation of the items or behaviours; guardians not noticing behaviour during previous veterinary visits, and; time since last veterinary visit. The present study did not include dog location, and so the impact of cultural differences in dog ownership and veterinary experience are unknown. Further, the ability of guardians to accurately identify fear in their own dogs is questionable [9, 63, 64]. Indeed, while Flint et al. [64] found training in recognising fear in dogs resulted in guardians being more likely to correctly identify mild/ moderate and high/extreme fear, they observed no corresponding change in reporting on the guardian’s rating of their own dogs. This calls to question the accuracy of guardian reporting of fear within C-BARQ, but also highlights the need for further investigation into what is required to ensure guardians are able to accurately identify fear in their own dogs. Conversely, while the current study’s sample size is large, it may reflect responses from guardians that actively seek to know more about their dog’s behaviour, and so, may represent responses from those who are more aware of their dog’s fear or body language. Further, we suggest that the proportion of dogs exhibiting fearful behaviour in the context of unfamiliar situations may be over-representative of experience in the veterinary clinic, as guardians may be reporting on fearful behaviour that occurs in unfamiliar circumstances outside of the veterinary clinic. Future research into dog experience in the veterinary context should corroborate C-BARQ responses for dogs who have recently visited a veterinarian with physiological measures of fear or distress and objective observations.

Overall, it is important to emphasise that the proportion of dogs negatively experiencing their veterinary visits is likely to be under-represented by C-BARQ respondents. The items (fear of veterinary examination and fear of unfamiliar situations) within C-BARQ explicitly reflect fear responses only, with no corresponding items for aggression. Aggressive behaviour in the veterinary clinic is also a very real risk for dogs distressed during their veterinary care [67]. While several aggression items do refer to grooming or handling by an unfamiliar person (Q14, Q21), they do not expressly mention the veterinarian or veterinary clinic and so were not included in analysis in this study. As such, guardian responses only reflect fear in the veterinary context and it is highly likely guardians with dogs that behave aggressively at the veterinary clinic are not represented in the proportion of dogs experiencing distress during veterinary examination or in unfamiliar situations.

## Conclusion

The results from the present study indicate that around half of companion dogs are experiencing some level of fear when receiving veterinary care, including one in seven dogs that show severe or extreme fear. The dog’s breed group, the roles or activities they have been involved in, where they were purchased from, their weight, the age of other dogs in the household and the guardian’s level of experience owning a dog accumulate to predict approximately 7% of the variation of fear observed during veterinary examinations. The same factors group together to predict 5% of fear of unfamiliar situations, 9% of touch sensitivity and 8% of non-social fear. While these factors play an important role in determining dog experience in the veterinary context, it is likely that other influences, such as the environmental set up of the veterinary clinic, history or past experience at the clinic, and the human-animal interactions (of guardian and veterinary staff), determine whether a dog shows fear at the veterinary clinic. It is important that the cause of fear in dogs visiting veterinary clinics be explored in more detail. For example, determining whether fear is a response to a previous negative experience in a clinic, or whether veterinary clinics are inherently stressful will help inform strategies that reduce distress during veterinary visits. Further investigation of how an individual dog’s background or the current veterinary environment combine with these risk factors is essential to bettering our understanding of a dog’s veterinary experience and in contributing to the continual improvement of dog welfare in the veterinary context.
